# Assessing Pain Research: A Narrative Review of Emerging Pain Methods, Their Technosocial Implications, and Opportunities for Multidisciplinary Approaches

**DOI:** 10.3389/fpain.2022.896276

**Published:** 2022-06-02

**Authors:** Sara E. Berger, Alexis T. Baria

**Affiliations:** Responsible and Inclusive Technologies Research, Exploratory Sciences Division, IBM Thomas J. Watson Research Center, Yorktown Heights, NY, United States

**Keywords:** pain, ecological momentary assessment, language, visual reports, Internet of Things, neuroimaging, machine learning, multidisciplinary

## Abstract

Pain research traverses many disciplines and methodologies. Yet, despite our understanding and field-wide acceptance of the multifactorial essence of pain as a sensory perception, emotional experience, and biopsychosocial condition, pain scientists and practitioners often remain siloed within their domain expertise and associated techniques. The context in which the field finds itself today—with increasing reliance on digital technologies, an on-going pandemic, and continued disparities in pain care—requires new collaborations and different approaches to measuring pain. Here, we review the state-of-the-art in human pain research, summarizing emerging practices and cutting-edge techniques across multiple methods and technologies. For each, we outline foreseeable technosocial considerations, reflecting on implications for standards of care, pain management, research, and societal impact. Through overviewing alternative data sources and varied ways of measuring pain and by reflecting on the concerns, limitations, and challenges facing the field, we hope to create critical dialogues, inspire more collaborations, and foster new ideas for future pain research methods.

## Introduction

In 2020, the International Association for the Study of Pain (IASP) updated its over 40-year-old definition of pain, officially conceptualizing it as “an unpleasant sensory and emotional experience associated with, or resembling that associated with, actual or potential tissue damage.”^1^ Along with this definition, six key elements were added to further contextualize the experience of pain, including: the simultaneous influence of biology, psychology, and society on pain perception, pain's differentiation from nociception, pain's learned elements, pain's ability to be both adaptive and maladaptive, that subjective pain reports should always be respected, and that there are multiple ways to show or be in pain. While terminology updates may not seem monumental, these changes were seen by many as crucial in moving pain research forward, representing a field-wide consensus of just how multifactorial, bidirectional, and personal the experience of pain is, something that had been debated, contested, or ignored for years (1–4).

Yet despite this acceptance and a growing commitment to studying and treating pain more holistically, the pain field is still relatively fractured, with scientists and clinicians largely remaining within and relying on their domain expertise and utilizing practices and techniques which don't always reflect the multidimensional or nuanced nature of pain. This dearth of collaboration and the continuation of disciplinary siloes is detrimental and ultimately unsustainable in the context in which the field finds itself today. Perhaps foremost in mind is the ongoing COVID-19 pandemic, which not only subjected millions of pain patients to postponed procedures, reduced access to medications, and exacerbated pain levels (5–9) but also further laid bare the rampant systemic inequity within many healthcare systems, effects which were compounded by discrimination within pain management more specifically^2^ (10, 11). Simultaneously, the speed of translating methods and data into digital formats is increasing not only the amount of data gathered but also the diversity of data streams collected—there is a need to be able to makes sense of all this information quickly to share insights and scale associated innovations in pain management. In turn, machine learning and other data science methods are increasingly being relied upon both in basic research and clinical settings. Together, the rising adoption of digital infrastructures, an increasing reliance on machine learning and big data, and an existing backdrop of the global pandemic and ongoing pain care disparities all necessitate a fundamental change in the ways we conceptualize, measure, monitor, analyze, and manage pain, as well as how and where we collaborate across research and care efforts.

It is with this in mind that we write this review. As researchers in the field of pain neuroscience and responsible technology, we ask—what does it mean to conduct pain research conscientiously and to create accountable pain science and pain-related technology that produces impacts which are minimally harmful to society's most vulnerable and mutually beneficial to the multiple constituents involved. We hold the responsibility to not only educate our fellow researchers and clinical colleagues on the technical advancements in existing and emerging pain methods, but also on their limitations and potential negative consequences. In this way, the paper is as much a call-to-action as it is an overview of pain research. We first summarize the state-of-the-art across emerging or strengthening areas of pain methods. Within each area, we describe the different data signals and sources that attempt to measure various aspects of the pain experience (biological, psychological, social) at different times, in different places, and for different applications. Next, we touch on protocol, technical, and human-centered considerations for each area, highlighting the critical assumptions or foreseeable ethical, legal, or social issues (12) surrounding the methodological advancements or associated technologies. We conclude by reflecting on the possible implications of these techniques given their overlapping strengths and weaknesses, and identify places where multi-, cross-, inter-, or anti-disciplinary approaches and collaborations could provide novel solutions, generate new research findings, and/or create new standards of care or practice.

## State-of-the-Art in Pain Methods

The following represent state-of-the-art (SOTA) methods that are either emerging in pain research or are producing new findings and foundational discoveries in the pain field. Importantly, we aim to include methods that *together* encompass the multidimensional nature of pain, from the biological and physiological aspects (neuroimaging and physiological sensors) to the psychological and cognitive aspects (self-report and language) all the way to the social and ecological aspects (visual reports and environmental sensors), finishing with machine learning as an overarching method that can analyze the different data types generated. This review is by no means exhaustive and does not include critical findings or research in the areas of sociology, anthropology, or epidemiology, which we consider greatly important to the pain field but outside the scope of this paper. Additionally, the methods reviewed were chosen based on the authors' previous research experiences, publications, and subject matter expertise. For a high-level summary and comparison of some of the key considerations across all methods, see Table 1.

**Table 1:** Summary of considerations across each reviewed pain methodology.

	**Considerations**	**Methods and signals**				
		**Patient-reported outcomes**	**Language proxies**	**Alternative visual reports**	**Physiological and environmental sensors**	**Neuroimaging**
Dimensionality	Patient pain expression is more open or unlimited					
	Captures short-term pain dynamics					
	Captures long-term pain dynamics					
	Captures multiple dimensions beyond intensity, quality, or location					
	Data has ecological validity (supports collection/analysis in multiple environments)					
	Data form is intended for pain-specific uses					
	More direct access to biological signals					
	Can more easily account for social influences					
Data	Requires active participant engagement					
Collection	Supports passive data collection					
	Internet/Connectivity/Smart Device-dependent					
	Data collection methods are scalable					
	Relatively easy to set-up data collection methods					
	Requires extensive researcher/clinician training					
	Prone to noise introduced from technical interfaces or environment					
	Prone to noise due to methodological and/or user errors					
Data Analytics	Can be qualitatively analyzed					
	Can be quantitatively analyzed					
	Data analysis is scalable					
	Requires advanced statistical analyses or complex processing					
	Existing analytical standards or benchmarks for reference					
Accessibility	Flexible, participant-tailored collection methods possible					
	May reduce patient burden (time, cost, or physical reqs)					
	May reduce researcher burden (time, costs, analysis reqs)					
Utility	Method used in clinical settings or contexts to aid in therapeutic decisions					
	Method itself can be therapeutic					

*Color indicates extent to which each consideration applies. Orange—generally true of this methodology; blue—generally not true for this methodology; grey—varies often; and black—not applicable or unknown. Machine learning as a technique is not listed here as it requires data from the remaining methods and signals. Additionally, the problem of introducing human bias into data collection and analysis is also not listed here, as it's a consideration that applies to all methods and varies greatly*.

### Reporting Pain—Self-Reports and Patient-Reported Outcomes

Patient reported outcome measures (PROs, PROMs, or self-reports) are often considered “the gold standard” for measuring acute and chronic pain. They include familiar condition-agnostic questionnaires, such as but not limited to the numeric pain rating scale (NRS or NPRS) (13), the visual analogue scale (VAS) (14), the McGill Pain Questionnaire (MPQ) (15), various versions of facial pain scales (16), the Gracely Pain Scale (17), and the Brief Pain Inventory (BPI) (18), as well as subsets of questions from much larger batteries like the National Institutes of Health Patient Reported Outcome Measurement Information System [PROMIS] (19). There are also PROs that focus specifically on designated pain conditions, such as the Western Ontario and McMaster Universities Osteoarthritis Index (WOMAC) for arthritis (20) or the Oswestry Disability Index (ODI) for low back pain (21). There have already been numerous reviews and comparisons of these scales (22–26). Rather than repeat these efforts, we focus instead on methodological considerations for their implementation, such as *where* and *how* they are administered.

Until recently, many PROs have been implemented cross-sectionally, at clinic or research visits, occurring every few weeks to months or more infrequently on the order of years. Their administration has also largely been limited to written or verbal responses (e.g., completing questions on a physical form or answering aloud in front of a staff member); this might happen once during a visit or several times in succession as part of provoked pain paradigms, psychophysics experiments, or quantitative sensory testing (27–29). This infrequent in-person approach might make sense for specific pain conditions or highly-controlled research questions but does not easily measure pain fluctuations (30) or pain perturbation by environmental, psychological, or socioeconomic factors (31–34). Likewise, many existing PROs are unidimensional or only focus on preconceived features of importance in the pain experience (e.g., pain intensity, pain location, or pain qualia), even though quality of life improvements may be higher priorities for many patients (35). In the instances where sleep, mobility, sociability, mental health, social support, or sexual health are measured, they are often collected as *separate* reports, creating much longer batteries, a reaction which might increase response burden or administrative burden (36–39). Finally, the context of administration can greatly influence self-report reliability—the environmental setting (in a clinic or research center), social setting (in front of a clinician or researcher), and temporal requirements (retrospective summaries or momentary one-offs) can all introduce a variety of cognitive and recall biases into pain reports (40). It is well-known that there are discrepancies between momentary pain assessments and pain memories^3^ (41, 42) due to heuristic strategies like the peak-end rule and cognitive phenomena like the recency effect. Additionally, patients' incentives, cultural display rules, social desirability, and confirmation bias can all introduce intentional or unintentional over- or under-reporting of pain dimensions (43–46).

To improve pain reports, recent methods have focused on increasing the frequency, diversity, and contextual validity of measurements *via* the use of ecological momentary assessments [EMAs (47)], where PROs are collected semi-continuously from a patient's natural environment as part of their day-to-day routines (48, 49). This kind of sampling can be done relatively easily due to the nearly ubiquitous use of internet, smart phones, tablets, and computers, as well as the implementation of apps and electronic data capture systems that seamlessly integrate cloud storage, content repositories, security measures, and data collection methods. Although EMAs have been used for a number of years within the healthcare space (50, 51), we have seen a recent uptake in these methods due to their relevance, utility, and need during the pandemic, especially in the realm of pain (7, 52, 53). Researchers have now been able to better study the temporal dynamics of pain (54), as well as find more nuanced relationships between pain intensity, location, descriptions, and flares with sleep disruption, psychological symptoms, personality characteristics, and concomitant activities or medication use (1–4)^4^. From a scientific and clinical perspective, care providers and researchers can more efficiently track their patients longitudinally without the need for increased visits (reducing care burden and study costs), and likewise, patients can participate in studies, clinical trials, and pain treatments more easily, reducing financial and time commitments by not traveling to on-site locations at designated or restrictive times.

While some of the in-clinic recall biases mentioned above are minimized or avoided using EMAs, biases related to reporting due to incentives (e.g., qualifying for treatment or passing eligibility criteria) or social desirability can still inflate or deflate self-reports, and cognitive or psychological elements such as current mood can still influence how a person rates their pain in a moment (55). Likewise, prior experience with self-report measures, patient preferences, and clinical history can all influence how people complete momentary assessments at home (40). Moreover, new biases or cognitive phenomena can also be introduced using this method depending upon implementation–studies have shown that the simple act of rating pain every day or receiving feedback about self-reports can change the qualities of pain time-courses and treatment responses due to increased awareness, reporting fatigue, habituation, or increased reporting accuracy [for one example, see (56)].

#### Protocol and Technical Considerations

Issues of compensation and accessibility are important to consider with any kind of PRO. At in-person visits, patients are often compensated for their time filling out these questionnaires and completing study requirements, as well as ideally reimbursed for travel. In contrast, there might be assumptions that EMAs require less work or less time due to decreased travel or increased convenience. However, at-home methods might be *more* intensive, since patients might be expected to rate more frequently or consistently within a given time frame, and they could also be perceived as more invasive from a privacy perspective. At-home PROs also have considerably more accessibility requirements, and there might be considerable upfront costs or training to implement an EMA system in a way that serves as many patients as possible (47). For example, patients need to have access to a computer or smart device, as well as internet or a data plan. If any or all of these are not available, teams must decide whether it is monetarily feasible to provide participants with a smart phone and data plan at no cost to them for the study duration. Additional flexibility in terms of user interface design or content collection may need to be implemented, particularly for patients who may require bigger text, read-aloud functions, or different UI interaction methods (e.g., voice control or buttons instead of sliders to accommodate hand mobility differences) (57). Adjusting frequency or time of PRO deployment or reporting to better fit with participants needs might be necessary to accommodate work and family time (58).

#### Human-Centered Considerations

It is well-documented that self-reports of pain are influenced by and change significantly based on participants' gender, age, ethnicity, and culture (44, 59, 60). For example, depending on age, previous experiences with pain, and communication ability, utilizing scales with numbers and words alone may not be the best way of measuring patients' pain. In lieu of this, pain drawings—which show the front and back of the body in its entirety—may be a better option for PROs as they allow people to visually indicate where on their body they are experiencing pain. These include simple pain maps where a person circles areas of the body or shades certain dermatomes or myotomes (61, 62), as well as more layered versions that incorporate colors, numbers, or words to indicate intensity and qualia (1–5). Importantly, body maps can be used longitudinally (63) to show how pain somatotopy fluctuates in time, with efforts to digitalize these into EMAs of their own (64). Similarly, the use of pictograms to illustrate pain experiences and sensations has also emerged—these consist of static images or cartoons that often show a person's facial expressions along with body language and additional context about pain location or source, and they have been helpful in studying pain in children as well as those with literacy, speech, or cognitive differences (65–67).

#### Critical Assumptions

Across nearly all numeric PROs measuring intensity, there is a discrepancy between the underlying neurophysiological pain response and the outward reporting of pain. While there is ample evidence that human perceptions of pain and pain relief are *not* linear—e.g., in cases like wind-up, hyperalgesia, offset analgesia, temporal summation, or sensitization (68–72)—the large majority of PROs essentially force people to translate this complex non-linear perception onto a linear scale. From a technical perspective, this limitation might be partly addressed *via post-hoc* statistics or a-priori models using non-linear functions. However, this may also represent a foundational disconnect within pain research and methods more generally. At the very least, it likely points to a need for a re-designed way to assess even the most fundamental dimensions of pain, like intensity^5^.

PROs have been and continue to be a fundamental research tool in pain methods, as they allow for a relatively easy way to communicate various aspects of the pain experience across different time scales and potentially across different contexts. Due to their prevalence and long history of use in the field, many also have a rich literature base and existing set of standard practices and benchmarks [e.g., minimal detectable change (MDC) scores, international population norms, etc.] that researchers can draw upon for comparison and analysis. However, as others have cautioned (73, 74), they should not be placed on a methodological pedestal as somehow being better or less problematic than other methods overviewed here. Critically, the reliance on numbers, pre-selected word anchors, and forced descriptions of qualia or intensity can change how people report their pain (75) and/or profoundly limit patients' ability to share their unique pain stories and define what pain is like for them (76). To measure pain more authentically, we need to understand pain using the person's own language, relying on patients' voices and narratives to define and extract meaningful information about their experiences.

### Listening to and Reading Pain—Language as a Proxy of Personal Pain Experience

Elaine Scarry said, “Physical pain has no voice, but when it at last finds a voice, it begins to tell a story” (77). In the last decade, the utilization of written or spoken language as a signal of underlying physical and mental health, indicator of neurological or cognitive conditions, or metric to quantify or categorize neuropsychological states has gained momentum. Previous studies have shown language's utility in identifying ingested drugs and dosages (78), quantifying depression severity (79), dissociating and predicting mental illnesses (80–82), and tracking changes in neurological disease symptoms (83–85). Given many chronic pain conditions are also neurodegenerative (86) and linked to changes in cognitive functioning or emotional processing, researchers are investigating whether quantitative language features can also measure pain qualities, although qualitative research investigating pain and language has been around for some time (77, 87–90). Unlike questionnaires, collecting patient language about their own experience is far less limiting, and whereas numeric PROs could be influenced by various cognitive processes, voice acoustic properties and lower-level linguistic and syntactic structures are harder to consciously influence in the same way (91, 92).

Prior work investigating the relationship between speech characteristics and pain have shown some promising links, primarily in the domain of acoustics and pain intensity. Breathiness (93), Mel frequency cepstrum coefficients (MFCCs) (93, 94), loudness (94), fundamental frequency (95, 96), formants (95), jitter (95), and speech rate (97) have all been associated with induced acute, perturbed, simulated, and/or chronic pain intensity in laboratory settings (98). Additionally, a number of content-related features from spoken and written language have also shown potential in assessing pain intensity, emotional pain severity, pain-related emotions, and changes in perceived qualia; these features have included personal pronoun usage, sentiment, verbosity, and psycholinguistic structure of words (99–106). Other aspects of the pain experience—such as quality of life metrics, pain interference, and even diagnostic category—have been evaluated using higher-level linguistic features, such as metaphor usage (107–109). Quantitative language metrics like these might be able to serve as an alternative to numeric ratings or as “proxies” of pain intensity or quality. This might be useful for both patients and pain practitioners—it could increase the number and frequency of pain data collection points (since many people talk throughout the day as part of their normal interactions) while simultaneously reducing collection burden (by decreasing the number of pain ratings needed).

Researchers have recently shown language's utility in understanding and measuring a variety of dimensions of the pain experience outside of qualia or intensity. Quantitative language features from semi-structured interviews have been used to quantify placebo response in chronic pain patients (110), and large-scale text-mining of electronic medical records has been utilized to detect pain disparities in underserved communities (111). Similarly, social media posts have been analyzed to longitudinally track patients and identify new pain phenotypes (112), geospatially monitor and characterize opioid use (113), conceptualize how pain is socially communicated (114, 115), track local and federal pain treatment policies (116), and discover population-level increases in pain conditions and symptoms (117).

#### Protocol and Technical Considerations

To capture acoustic properties of language, speech is required. Speech can be elicited actively—*via* responses to short narrative or monolog free-speech prompts^6^, dialogue and semi-structured open-ended interviews (118–121), descriptions of pictures (122), reading passages (123), or verbal motor tasks like phonoarticulatory diadochokinesis (124)—or passively, by randomly sampling voice snippets from the environment (125, 126). Speech data can be recorded using professional sound equipment or with simple hand-held recorders, as well as *via* built-in microphones in phones and computers. Alternatively, one can also collect non-spoken language or transcribe collected speech into text—in these instances, participants might be asked to write or type their responses. They might also consent to let investigators use prior or on-going free text in the form of social media posts or direct messages, data which can also be obtained *via* publicly-available datasets, licenses, or crowd-sourcing (127).

The methods chosen and features calculated need to be designed with the specific application or pain condition in mind, as many factors will influence their utility and feasibility. For example, open-ended monolog prompts might be appropriate for adults with pain but may not be easily understood or fully answered by children; similarly, for some populations, speech may be easier or more natural to elicit than typing a response, which might be better for participants who text often or for participants who might find texting more convenient than talking during the workday. Likewise, the use of off-the-shelf or personal recording devices might make sense for larger clinical trials or at-home assessments to scale data collection and analysis, but for smaller studies interested in vocal characteristics of pain, investing in recording equipment and sound-proofing might result in higher quality acoustic data. Additional study design choices could also dictate how burdensome methods like these could be for patients—outside of elicitation methods and frequency, elements such as randomization without repetition and varied prompt or picture topics might also need to be employed to sustain engagement and reduce boredom [for some examples, see (128)].

There are also many procedural and analytic considerations. Hand transcription of voice responses and qualitative coding of language using methods like grounded theory (129) or interpretative phenomenology analysis (130) might work well for smaller studies, but these approaches are not always feasible for larger studies—they may not scale with data collection or analysis needs, nor are they easily reproducible across studies or applicable for acoustic designs. If participants are collecting language using at-home methods, there will likely be additional sources of noise (e.g., additional voices, background TV, movement) (131), which affects transcription accuracy and acoustic feature calculation. These methods will require participant training to minimize these effects, as well as various pre-processing software to filter out these interferences (132–134). In the absence of paid-for transcription services, researchers can also utilize Automatic-Speech-Recognition (ASR) to transcribe spoken language into text for easier data processing (135). However, ASR's transcription quality is only as good as its underlying acoustic models and associated training data (136). Reducing gender and racial biases in ASR and large language models is an area of on-going investigation^7^ (137, 138); instead of relying strictly on population-based language models for transcription, practitioners can pre-train models on small samples of transcribed individual speech from reading passages [e.g., the Rainbow Passage (139)] to improve accuracy and reduce word error rate (140). In addition to issues of scaling collection and transcription, researchers will also need to think about how to scale analyses of such a rich data source. For this, natural language processing (NLP) (141, 142) is commonly used, deploying sub-methods like named entity recognition or topic modeling to classify differences in pain (143–145).

#### Human-Centered Considerations

The language we use to represent pain changes as a function of our neuropsychological development, our experiences with pain, and relatedly with age (146–148)—findings or methods that hold true to one age group may not be applicable to another. Similarly, gender identity, associated norms or social conditioning, and racial or ethnic community practices and values can also influence if and how people talk about pain (149–153). Likewise, culture, religious beliefs, and native language also influence how pain is verbalized, described, and conceptualized (154–162). All these elements can affect patient engagement with the method itself, as well as downstream analyses, interpretations of the data, and validation of any results.

#### Critical Assumptions

Utilizing patients' language as the “ground truth” is in line with the basic premise that pain is inherently subjective and highlights the necessity of respecting patient's reports. However, the utilization of patient language holds normative and even ableist assumptions about the best forms of communication or the legitimacy of different communication sources. There may be some people who prefer not to communicate through talking or may be limited in their ability to use written or spoken language due to cognitive differences or neurodivergence more broadly. There is some research showing existing PROs and language-based measures fall short for people with Autism or Asperger's who may communicate pain differently^8^ (163). This is particularly troublesome given this community may experience more acute and chronic pain than other members of the neurodivergent community or their neurotypical counterparts. Additionally, many of the language models used today carry assumptions about the acoustic tones and rhythms found in “typical” conversational speech. This could negatively impact pain patients with speech impediments (164) or those whose language patterns do not match that of model creators (typically White, English-speaking men) (165, 166)^9^, and could exclude or delegitimize the use of lyrics, rhymes, or singing, all of which can also be used to communicate pain (167).

Thus, although the recent use of language proxies for pain have been an important development in the field—allowing for more intuitive, social, and patient-centric measurements of broader experiences in ways that are relatively flexible and often scalable—there is still work to be done to make this method inclusive. Moreover, sometimes language as a signal simply is not enough, as many authors have already eloquently described; for example, Virginia Woolf said, “…let a sufferer try to describe a pain in his head to his doctor and language at once runs dry (168)” and Emily Dickinson said, “Pain has an element of blank—it cannot recollect when it began or if there were a time when it was not” (169). In moments when pain is particularly intense or unrelenting, people in pain might not be able to speak. Additionally, non-verbal utterances like cries, gasps, or groans may be produced instead of words, and methods and techniques which can both capture these vocalizations and dissociate them from other noises and language features would be useful and appropriate (170).

### Seeing Pain—Use of Visual and Alternative Pain Reports

As an alternative to quantitative or language-based assessments, there are also self-reports which try to bypass traditional forms of reporting (or use words and numbers sparingly as additional components to the primary report, which is *visual* in nature). While face scales, pictograms, and body maps mentioned previously might be considered visual reports to a certain degree and are important from an accessibility standpoint, they have pitfalls surrounding representative assumptions as to what base bodies look like (e.g., average size with two legs and two arms) with no way to easily account for physical differences due to weight, genetics, or corporeal disruptions from illness or trauma. Likewise, facial scales have assumptions of what reference faces are comprised of (e.g., two eyes, two ears, a nose, and a mouth) and what base facial expressions look like. They also reiterate beliefs about the existence of universal expressions or emotions (171, 172)—an idea that is still debated (173, 174)—as well hold assumptions that everyone can intuitively understand facial expressions or easily infer meaning from them, which isn't true (175). In this section, we focus on different methods that try to visually depict aspects of the patient's pain experience, driven primarily by their embodied and situated perspective.

Visual arts have long been utilized in community and healthcare spaces as therapeutic resources, places of resistance to the biomedical model, and forms of communication and community building (176, 177). Visual art can take many forms—painting, drawing, handwork (e.g., pottery, sculpture, sewing, or basket weaving), movement, cinematography, photography, and acting. Many if not all these genres have been explored in the context of pain's physical and psychological forms (178–180), and there are an increasing number of research studies and grassroots efforts aimed at utilizing self-created art to depict and share patient's subjective pain experience. For example, there have been methods that ask patients to paint or draw what their pain looks or feels like as a form of expression (181–183); there are also online communities of pain patients who collect and showcase art related to their pain, with the specific goal of improving communication with care providers^10^. Others have used photography and mixed media to capture various aspects of people's pain (76, 184), as well as clay work and sculptures (185) and patient co-created animations (186, 187)^11^. There is also evidence of the ability of visual arts to communicate aspects of pain in theater and performance settings (188), particularly in the realm of dance and movement. Research in dance-movement therapy (DMT) is beginning to show that certain kinds of movement-based exercises can communicate pain in new and beneficial ways. For example, the DMT exercise called “mirroring” asks patients to communicate their pain through movements or postures, during or after which they then visualize these movements mirrored back to them by another participant (who might be another pain patient, a therapist, a loved one, or care provider). Semi-structured open-ended movement displays like these have been shown to improve empathy toward people in pain, transfer bodily understanding and awareness to those not experiencing pain, and potentially cause analgesia^12^. Similarly, there has also been an increase in the use of cinema, film, video recordings, and documentaries to try to communicate multiple dimensions of pain (and do so multi-modally through stories, art, and depictions of people in pain and their environments)^13^. Early qualitative research has shown that viewing films of pain or pain performances may impact how care providers think about or conceptualize their patient's experience (189). Methods like these have been able to elicit elements of pain not typically asked about in the clinic, on questionnaires, or within interviews such as feeling disconnected from one's body, a loss of identity, feeling powerlessness, or fuller senses of suffering^14^ (189, 190). They have also been transformed into re-usable and clinically-impactful tools that patients can use to have more personalized conversations with their care providers (75, 186).

The use of visual or alternative pain reports like these is particularly exciting for a couple reasons. First, they challenge the pain field to consider pain displays that are not only defined by an individual in pain but also by larger communities experiencing pain (shifting ideas about self-reports to *community*-reports). They also encourage the pain field to consider the use and benefits of participatory methods whereby patients co-design or even dictate what pain measures and metrics look like, in some ways challenging the notion of what it means to be a “pain expert” or pain researcher (191). Second, they open the door to novel pain measurement tools that could be back-translated into clinical use *via* converting traditional or language-based PROs into something visual. For example, clay has been used to sculpt expressions of emotions or re-communicate body language (192)—what would this look like in the context of PROs that use faces or body maps as primary pain measures? Could patients use clay to show how their bodies feel, where the pain is located, and/or what the consequences of pain are? Similarly, using visual or mixed methods approaches in body map contexts might be able to capture pain elements more intuitively and include those *outside* of intensity or location, such as pain directionality up or down, pain radiation outwards, or pain fluctuation from a source. What would it look like to visually record the completing of static pain drawings in real time as a method to measure the temporal evolution of multiple aspects of spontaneous or on-going pain? More investigation is surely warranted in being able to translate, validate, or mix visual and non-visual reports and methods.

#### Protocol and Technical Considerations

Because these forms of expressing pain rely on patient-specific communication and can also be time-intensive to create or perform, it makes it difficult to scale or re-apply data collection methods across different populations, cultures, and even across sample sizes of patients (like in the context of larger clinical trials). Likewise, the subjective and intensely personal nature of these methods creates tensions between the need for unique qualitative data and interpretations on the one hand, and current scientific, technical, and medical requirements of reproducible data and quantifiable results on the other. It's challenging to be able to validate these methods across new patient cohorts or new contexts, and even more challenging to form quantifiable measurements of the data (and do so in ways that don't reproduce the pitfalls of other methods or exploit, reduce, or ignore patient experience). While there are a lot of unknowns in this realm, we note that this line of inquiry remains essentially limited to the social sciences and relatively unexplored in the biomedical pain sciences. It would be worthwhile forming and funding interdisciplinary research collaborations between quantitative and qualitative experts, such as but not limited to pain scientists, clinicians, social scientists, artists, and patients.

#### Human-Centered Considerations

The visual methods summarized here all try to center the human-experience of pain as expressed by individuals or even collective communities. On their own or in certain therapeutic environments, they can be empowering and disrupting. But within the context of the current healthcare system complex and under the biomedical gaze, they can still perpetuate harmful stereotypes and assumptions, namely that pain is always shown or eventually can be “known” or made obvious (189). Notably, this assumption is found across *all* the methods reviewed here and is not unique to visual reports, although they illustrate it well. The reliance on appearance or the presumed outward expression of pain in some contexts can function as a form of forced assimilation, as it does not always acknowledge the hidden aspect of suffering and ignores that many pain patients are forced or prefer to hide their pain at work or at home, or present as “normal” in order to function in society [a medical form of “passing” or “covering” (193, 194)]. Due to the assumption that significant or severe pain will eventually be “seen,” there are often incongruences between verbal reports, elicited behavior, and felt internal reality which might cause biased pain assessments or withheld pain treatments. As a community, pain patients already must deal with numerous stigmas surrounding hidden disabilities, perceived burden and guilt, labor and productivity norms, and assumed malingering (195–200). Care should be taken to reduce, minimize, or prevent (if possible) additional stigmatization through the method used or data collected.

#### Critical Assumptions

Alternative visual reports have played an important role in the pain field, as they have not only allowed for fuller expressions of the pain experience but also largely challenged the traditional status quo of “acceptable ways” to communicate pain. However, there are numerous instances where pain cannot be verbalized, displayed easily, or even “known” at all due to injury, disease, age, neurological difference, or cognitive impairment (e.g., in advanced stages of dementia, in minimally conscious individuals or comatose patients, or in those with severe communication difficulties from ALS or locked-in syndrome). There exist clinician-reported outcomes of pain (CROs) (201, 202) as well as third-person reported outcomes (203–205), which ask care team members, providers, and loved ones to rate their perceptions of the patient's pain. However, these are fundamentally limited since there might be no way for a patient to concur with the pain assessment, advocate for themselves, or rebut care decisions. Additional measures which don't rely on self-reports, self-displays, or others' reports of pain may also be useful and clinically relevant.

### Sensing Pain—Measuring Pain Through Physiological and Environmental Sensors

The multidimensional nature of pain lends itself to being studied multimodally using various body, environmental, and ambient sensors (206). These are often part of a much larger system of wireless and connected devices that collect, transfer, store, and analyze data over a network (commonly referred to as the Internet of Things, IoT). IoT and sensor-based methods can be used to collect more frequent and fine-grained assessments within a growing digital health ecosystem (207). As with EMAs, these tools may reduce some of the reporting or cognitive biases found in clinical or research settings and may better contextualize pain within a person's natural environment. These methods also have the potential to reduce patient burden by not requiring frequent or active reporting, since many sensors can collect data entirely passively (208). Additionally, the use of sensors might make participating in clinical research more accessible—instead of incurring costs (time, energy, and money) to go to a visit, the clinic or research center is brought to the patient (208).

There are multiple studies showing physiological and environmental sensor utility for pain in “smart home” environments (209–212); importantly, many of these studies pair cross-sectional PROs, crowd-sourced reports, or longitudinal EMAs with sensor data (213). Some devices are used to measure specific aspects of pain sensation, perception, or psychology, while others are used to capture quality of life. The former typically fall under the category of body sensors, which capture physiological or biometric signals from the body. Wrist-worn devices and epidermal patches are often used to calculate heart rate variability (HRV), which has been correlated to acute changes in or sustained alterations to sympathetic and parasympathetic tone due to primary (214, 215), secondary (216), and chronic (217, 218) pain, as well as psychological stress (219).

Quality of life measures tend to utilize environmental or ambient sensors, although wearables are used as well. These might include passive infrared motion and magnetic door sensors to monitor movement and label certain “activities of daily living” (ADLs) based on room entry (e.g., cooking, bathing, entering/exiting the home, sleeping). Existing smart home devices like internet-enabled thermostats, humidity detectors, and light switches can also be integrated into data collection to better label certain ADL events (210, 220). Additionally, built-in device accelerometers can be used and integrated to monitor mobility *via* actigraphy, step counts, and time spent in different three-dimensional positions (221–223)^15^. Other wearable and environmental sensors are used to quantify activity levels (224, 225), gait, balance, or movement quality (226–230), and posture (231, 232), all of which might be applicable for a pain patient depending on their condition or at-home treatment or rehabilitation regimens (220, 233).

The use of sleep sensors have also been implemented, since pain itself and common pain medications like opioids are known to disrupt sleep patterns (234–236). Sleep sensors can take many forms (237, 238), including portable polysomnography sensor systems embedded into head caps, smart apps that capture movements or noise, infrared cameras that capture body position, or pressure platforms put under or into mattresses, pillows, or cushions (239–244). Finally, facial expression monitoring of pain intensity is also an active area of inquiry (245–249), part of a growing subarea of IoT and digital patient monitoring (237, 250) that utilizes multi-camera systems and video recordings to measure “emitted” facial expressions in real-time or retrospectively (251–253). These measures have been primarily based on automatic recognition of facial action coding system units (FACS) (254) and associated sentiment (255), although some analyze audio and visual signals together (256–259).

Nearly all these devices, methods, or signals have been used in critical, clinically complicated, or end-of-life situations. HRV has been used as an indicator of nociceptive pain in people who were minimally conscious or in a vegetative state (260), smart watches have been implemented to monitor movement and manage pain symptoms for patients in palliative care (261), EEG reactivity signals have been used to infer pain in comatose patients (262) and people with cognitive impairments (263), and pain facial expression has been measured in patients with dementia (264), intellectual or developmental disabilities (265), and infants (266). Although this review is focused on human pain methods, we also note that some of these sensor-based methods are also utilized to measure pain in animals, including automatic facial recognition (AFR) (267–270).

#### Protocol and Technical Considerations

When implementing sensor-based measurements, choosing which devices to use or program is not trivial, as there are hundreds on the market. Moreover, many direct-to-consumer devices claim to accomplish similar things or perform similarly to SOTA devices without openly shared data or validation studies (271–273). Additionally, many devices come with their own out-of-the-box or off-the-shelf output metrics that are automatically computed for the user or consumer; care should be taken as to whether or not to take these metrics at face value (since many calculations are “black box” or proprietary and thus cannot be easily examined for noise, privacy, reliability, or accuracy)^16^ (274, 275). Likewise, researcher access to raw, unprocessed data and researcher ability to pre- and post-process these kinds of large and noisy data streams need to be considered.

There are also many layers of complexity to implement multiple device collection congruently and privately, particularly if these methods are deployed outside of a clinical or research setting. The sensing system itself, the network connection, data communication and transference, data storage location and requirements (e.g., de-centralized or centralized), data management and security, applications, data standard compliance, and even data analytics (e.g., on the cloud, federated learning, or locally on approved devices) (207, 276) will all affect starting implementation and maintenance costs. Similarly, the reliance on technology for all measurements has some inherent risks for data collection—data quality, accuracy, detection rate, and obtainment can all be influenced by environmental, connectivity, and human factors such as: low light conditions, occlusion, power outages, wearing and charging compliance, fitting issues, or devices going out of network or range of upload hubs. Securing additional secondary methods for collecting data (e.g., diary methodology or questionnaires) should be considered, as well as backup device storage and queued data transfer abilities.

#### Human-Centered Considerations

Adaptability is an important design element to consider when deciding on devices and sensors to use. For example, wrist-worn devices need to have adjustable bands to be able to accommodate varying wrist sizes, and different materials should be considered based on skin sensitivities, skin fragility, or skin allergies; the same is true for patches and wireless EEGs, since adhesive materials can be abrasive or irritating to skin (277) or may not adequately capture signals depending on hair type or head fit (278). Patient living conditions and preferences should also be considered, since placement of sensors and co-use by cohabitants also matter (e.g., bed sensors may not be appropriate for people with partners, children, or pets that co-sleep with them, or for patients who sleep in non-traditional locations or environments) (279–282). Importantly, sensor methods might create new forms of participant burden (283, 284). Setting up devices for proper data collection and the need to charge devices might contribute to confusion or additional work for patients (285, 286)—having systems, methods, or devices which can reduce manual set-up, automate data uploads, or maximize battery life will aid in their long-term feasibility and engagement. As discussed with EMAs, there is also the presumption of access—considerations regarding interface design, digital literacy, and digital inequity must all be taken seriously. Time costs associated with patient or caregiver training, and monetary costs for scaled set-up at home or within a clinical infrastructure also should be accounted for up front. As with other methods, consideration of demographic and comorbid health conditions and abilities is also necessary. Gender, age, body mass index, baseline health, exercise regimens, and other factors can influence many of these physiological signals. For instance, HRV as a signal changes as a function of age, activity level, and sex (287, 288), and sensors or technologies themselves can also work differentially depending on patient variables including skin color (289, 290).

#### Critical Assumptions

Sensor-based measurements of pain are important resources for studying and assessing patients' experiences. Their contribution to the pain field has largely been their ability to collect multiple kinds of data through often passive or minimally burdensome methods, allowing for a much larger breadth of use and the discovery of correlations with other ecological events, physiological signals, or patient activities. However, these measurements also have the potential to perpetuate harmful suppositions about what pain “is.” For example, many of these measures likely follow the recent trend of calling pain a “fifth vital sign”—while pain perception and report should indeed be considered *vital* to a person's health and well-being, the comparison to a vital sign immediately conjures up mechanisms akin to strictly biological measures like heart rate and blood pressure (72). In some ways, a hyper or a singular focus on these kinds of metrics can work to erase the comprehensive and multidimensional conceptualization of pain that IASP has put forth. This framing ignores the intensely social aspect of pain communication by solely placing the data of interest as something emitted by the patient, as opposed to also being witnessed by others. Likewise, there is often an unsaid assumptions that sensor-based metrics are more objective or less noisy than self-reported metrics, and some even argue that they work to reduce the kinds of human biases seen when measuring pain (291). However, as mentioned, sensors are subject to numerous points of human and environmental interference that can introduce sources of noise. Moreover, we also know that the decisions of which kinds of sensors to use are not neutral (292) and that the underlying sensor data that accompanying algorithms are trained on are not representative. This is easily seen in automatic facial recognition, where pain inference accuracy remains questionable at best (293–295). Setting aside the technology and methods for a moment, we already know that humans aren't reliably accurate at recognizing the emotional expressions of others^17^ (172). This effect is seen not only in day-to-day life, but also concerningly within clinical settings, where the facial and bodily expressions of women and people of color are notoriously under-valued, minimized, or ignored altogether (296–303). These existing human errors and pervasive prejudices means that the data sources and the data labels that go into methods like these are in turn quite biased, as are interpretations of the results, which has implications not only for downstream use but also how to even define accuracy in the first place.

There are additional issues related to privacy, surveillance, and consent that are seen using these methods. While most of the data generated are biometric in a generic sense (generated by biology), some data can also be considered biometric in the legal sense (reasonably identifying of an individual), which raises important ethical and legal concerns surrounding protecting privacy, identity, and sensitive health data. This is particularly relevant for passive data collection methods, where someone might easily forget or not notice that a device is on and upload information they may not have intended or wanted to. In addition to privacy concerns, long-term psychological consequences of “the quantified self,” informed and flexible consent (including the right to be forgotten), transparency of data collection and use, and autonomy in decisions made and interpretations formed from these data are all critical issues to think about (304–311).

### Imaging Pain—Neuroimaging Measurements of Pain Sensation and Perception

Outside of wearable and environmental sensors, neuroimaging has also been used to study additional physiological and biological components of pain. There are various technologies for imaging and measuring brain structure and function as it relates to pain. Chronic pain research, in particular, has benefitted tremendously from these tools, strengthening evidence that acute and chronic pain mechanisms are distinct (312, 313) and shifting the field's focus of attention from somatosensory processing and “the pain matrix” (314) toward brain circuitry related to reward and decision making (30, 313, 315), emotion (316, 317), and memory (41). The novel findings and confirmatory evidence offered in these studies have fostered innovative research, and substantiated new targets for treating chronic pain with active pharmaceuticals (318) and placebo (319, 320).

Yet, there is still much to understand about the data created by these technologies. The physical properties of magnetic resonance imaging (MRI), for example, are well-known, but the physiological basis the signals detected is still an active area of research: cortical thickness (321, 322), synchronous activity in various brain circuits (323, 324), and white matter integrity (325) all change with experiences of pain, but the causal links remain hidden. Likewise, electroencephalography (EEG), nearly a century old technology, produces data whose source mechanisms are still not fully known: why does the brain produce specific oscillatory frequencies, what structures and processes account for those frequencies, and why do they change with pain? Of course, answering these questions could create fundamental shifts in how researchers understand and study pain. But in the meantime, analyses constantly evolve and adapt to account for changing knowledge and technological advances. Thus, while neuroimaging can produce exciting research findings and useful insights that may one day lead to more effective clinical pain treatments, there are several practical issues to consider.

#### Protocol and Technical Considerations

Each imaging modality comes with unique advantages and limitations. Temporal resolution is highest in modalities that more directly measure brain electrical activity (like EEG) and are much better at capturing fast neural events, but because these measures are most often taken at the surface of the scalp, detecting signals from pain-relevant deeper brain structures is challenging (326). On the other hand, fMRI is more suited to pain-relevant subcortical sites and has considerably greater spatial resolution, but signal detection is dependent on less-direct measures of neural activity and are orders of magnitude slower. Any interpretation about pain/neuronal relationships using this method must take into account blood oxygenation, hemodynamics, and other sources of physiological activity mixed within the signal (327). Electrocorticography (ECoG) has both high spatial and temporal resolution, as well as direct measures of neural activity, but it requires highly invasive surgery, and thus can only be used under special clinical circumstances (328). Additionally, each modality is susceptible to various sources of noise like breathing, heartbeat, eye and body movements, and electrical activity from the surrounding environment. These confounds may be accounted for using a range of signal processing methods (329–331), although there is no agreed-upon field standard.

The limits of these methods to specify and distinguish physiological and cognitive functions relevant for pain remain questionable, particularly for MRI. The “pain matrix,” for example, a highly studied brain circuit which exhibits coactivation of somatosensory, cingulate, and insular cortices with painful stimuli, has mostly been disregarded as pain-specific and is now more commonly recognized as a network which responds to “salient” stimuli, painful or not (314). Similarly, putatively more pain-specific and granular sets of brain regions (332, 333) have shown little to no improvement in distinguishing pain. A recent study showed that using only small fractions of these maps resulted in almost no loss in researchers' ability to decode perceptual pain states (334). Further, the general notion that high spatial resolution in fMRI is even capable of meaningfully explaining cognitive functions is being tested^18^. These results further support the need for cross-disciplinary research, suggesting that pain is too complex to be adequately understood using current neuroimaging methods. A harsher take might be that popular neuroimaging methods altogether cannot meaningfully describe fundamental functional properties of the brain (335), and that funding may be more appropriately allocated to methods with greater promise.

#### Human-Centered Considerations

Pain research necessarily involves some degree of vulnerability, and this is only compounded when imaging is involved. In attempts to establish experimental control, clinical pain studies are often less than inclusive, with exclusion criteria that may disqualify people with comorbidities, clinical or drug use histories, or who are subject to various other social circumstances. Imaging creates even tighter restrictions: experiments can be lengthy and require multiple visits at special facilities, which can make it difficult for those who need to accommodate work or dependent care schedules, and those without reliable transportation or adequate funds to travel. The imaging process itself can be physically uncomfortable for people who cannot stay still or in certain positions for long periods of time, or those who suffer from anxiety in closed spaces. For MRI experiments, magnetic material must be removed and/or absent from the body during scanning, which makes it impossible for people with certain implants, work histories or medical/cosmetic procedures, or those who prefer not to remove certain items from their body, to participate. Likewise, to obtain stronger EEG signals, researchers may choose to exclude people with coarse and curly hair in order to get closer contact between the sensors and the scalp (278). Enforcing all these criteria, of course, may leave researchers with findings that cannot be generalized outside of a select, homogeneous, subpopulation with limited pain experiences.

The clunky setup of many neuroimaging devices may also prohibit measuring everyday experiences of clinical pain. For example, research participants may not feel their pain while seated or laying in certain positions. Pain may be sporadic and unpredictable, which makes longitudinal tracking or early-stage detection of disease status difficult. Data collection sessions may also be too short to capture slow pain dynamics which can occur over hours, days, or longer. Attempts to evoke feelings of pain while collecting data may impose an unnatural pain experience, and reporting pain during data collection may also affect pain perception (336). For these and other reasons, current neuroimaging methods are not scalable and further limits those who may wish to participate in research. Mobile neurotechnologies may ease some of these restrictions^19^, but signal quality from these devices has yet to be fully validated by the research community.

Neuroimaging experiments can also range widely in financial cost. While EEG experiments are relatively cheap, the going rate for a single hour of MRI scanning may be hundreds to thousands of dollars. This price is compounded by a pervasive fascination with *big data*. “Scale thinking,” or the desire to process and own an ever-growing volume of work, not only frames how industry tends to think about its problems (337) but also influences the research community, as evidenced in efforts to conduct larger and larger neuroimaging datasets like the Human Brain Project^20^ and Human Connectome Project^21^. The discoveries and solutions yielded from these efforts, however, may not be proportional to the investment. In a recent simulation, increasing participants by orders of magnitude resulted in only incremental gains in predictive performance^22^. While not disqualifying, results like this one raise questions about what society should expect from these technologies, whether certain methods may reap larger benefits from scientific funding, and at what point these decisions should be made (and who should be making them).

#### Critical Assumptions

As mentioned, neuroimaging has been a vital method in the field of pain research, providing us with a non-invasive way to interrogate and better understand underlying neurological patterns in pain perception or chronification. Yet, while the brain is undoubtedly key to understanding the mechanisms of pain, neuroimaging researchers should resist promoting neuroessentialist views that reduce the pain experience to mere brain or spinal cord signals. Doing so risks diminishing the importance of measurement from other methods, may misdirect more practical efforts to diagnose and treat pain (338), and may proliferate a social dependence on scientific authority (339).

### Analyzing Pain—the Role of Machine Learning in Pain Data and Pain Research

Pain researchers in all the fields and methods presumably generate data in volumes or complexities that may become exceedingly difficult to manage. Machine learning (ML) can ease some of those difficulties, helping researchers to process data, as well as explain, predict, and understand various aspects of pain itself. By identifying patterns in data without relying on explicit programming or rules, machine learning allows researchers to further investigate those patterns which might not otherwise surface. Different aspects of data cleaning, clustering, or modeling can be done with machine learning, and it is becoming a standard tool across many scientific disciplines.

Most machine learning tasks come in two different forms, supervised and unsupervised. A “supervised” machine learning model may be designed to classify pain responses, attempting to determine a person's analgesic response likelihood (319) or the presence or absence of pain (340, 341), or it may be designed as a regression problem to indicate how a person's pain rating will change over time. This pattern-finding process is part of “training” a machine learning model, which ultimately shapes the model's statistical properties and its resulting output scores. “Unsupervised” machine learning, on the other hand, identifies patterns in data without the need for labels. An example of this might be to find predominant themes in text data from patient interviews, or for segmenting patient populations into different groups. Both supervised and unsupervised methods can be carried out using algorithms with varying degrees of complexity, each of which may be more appropriate than others for handling specific data types, dimensions, and quantities. While other sections of this review specify protocol or technical considerations for their respective methods, a full accounting of those considerations is beyond the scope of this section due to the breadth of algorithms available, and the nuances which may vary according to the kind of pain data being used. Importantly, comprehensive coverage of those considerations have been reported elsewhere (342–344). Instead, in this section we concentrate on the intersecting human-centered considerations and critical assumptions within machine learning, particularly around one binding requirement for all its forms: the need for a large amount of data. The growing incentive to generate pain data is a key reason for the growth of machine learning in pain research, and vice versa.

#### Human-Centered Considerations and Critical Assumptions

The healthcare industry generates data in tremendous volumes, and is projected to be one of the fastest-growing data producers in the world by 2025^23^. Various types of patient data attract machine learning researchers to healthcare, but the availability of clinical images in particular is pushing radiology toward the forefront of adopting and integrating machine learning into clinical practice (345–347). One recent study using convolutional neural networks (a popular kind of machine learning used for visual object recognition) demonstrated researchers' ability to identify non-standard patterns of knee pathology, and predicted patients' subjective pain reports 61% better than traditional measures (110). Additionally, using this non-standard approach, researchers were able to account for racial disparities in pain perception at 5x the rate than previously accomplished. This finding is particularly important considering growing concern around imbalanced community representation in medical data: it shows that machine learning can uncover pain-related pathologies to the benefit of marginalized communities, despite their historical exclusion in healthcare.

This is a rather *uncommon* result in machine learning literature, however, which typically results in an elevated risk for maintaining or amplifying existing societal disparities due to the systemic inequities under which data is created, collected, and used (292, 348). This problem is often framed in terms of *bias*—applying bias correction through data preprocessing methods^24^ (349) or adjusting model parameters through procedural fairness^25^ are then frequently suggested as ways to mitigate these risks. Bias, however, can affect machine learning at many stages of development and implementation, and this can be much harder to address with technical approaches which can't account for existing societal inequities and power imbalances (350, 351). An example of this is shown in a recent study that corrected a healthcare algorithm which disproportionately assigned Black patients lower risk scores, despite having worse health outcomes than White patients with the same score (352). Here, the old algorithm used a financial variable as a proxy for health outcome, which differentially corresponded to communities due to differences in needs, access, and trust in healthcare institutions (353, 354). A corrected machine learning model was created through more rigorous evaluation and the use of a dependent variable which more faithfully represented patients' actual health. The result was a reduced disparity of scores across racialized groups.

Of course, designing a machine learning model like this requires more knowledge than is available in a database. It requires a model evaluation process that extends beyond calculating conventional performance metrics, and an ability to critically assess the presumed impartiality of data. This is particularly pertinent in pain research, as pain is neither an objective measure, nor is it measured or treated equally across different patient and societal communities (300). Accordingly, attempts to objectively encode it for the purpose of scaling research or medical solutions through machine learning continues to pose a risk of entrenching societal harms (337)—more data does not guarantee more meaningful discovery or better predictions. Contextual knowledge of data is also needed, as is transparency about the analytics—without either, scientists can end up making the pain experience worse for patients navigating an already unfair system^26^.

This point is perhaps most germane to the open-source machine learning and data science communities who are known for a culture of sharing, while at the same time publishing highly controversial work in areas where data is plentiful, but subject matter expertise or critique is lacking. The quick surge of machine learning enthusiasts offering their tech expertise in the beginning of the COVID-19 pandemic is a prime example: in a study evaluating 232 COVID-19 predictive models, it was found that none of them were clinically useful, and almost all of them were “poorly reported,” had a “high risk of bias,” and reported performance that was “probably optimistic” (355). This result may be due to the conventional approach of judging machine learning model quality around benchmark metrics that often don't translate well in real-world practice (356, 357). Machine learning models built on the online availability of face data, for example, have spurred studies making claims about the ability to computationally model one's trustworthiness (358), sexual orientation (359), and criminality^27^, all of which are based on the debunked pseudoscience of physiognomy (360). Facial emotion recognition, as well, stands against long-held observations that emotion and affect are too complex to be captured by facial expression alone (172). Yet, facial expression data remains a valuable commodity for businesses offering affect recognition solutions^28^, as well as a popular data source for machine learning studies, including those attempting to measure pain (293, 361, 362).

However, open-source data sharing, and the communities that partake, can and do create positive opportunities for the pain research field (363). As of writing this paper, Kaggle, a popular data science challenge forum, had 964 notebooks under the search for “pain”^29^; GitHub hosts a list of open-source pain databases^30^; and OpenPain^31^ hosts the largest set of brain images from academic pain studies, available to anyone. The culture of sharing in machine learning presents relatively low barriers to those interested in studying pain (given sufficient time and internet access), opening the field to fresh enthusiasm and unique perspectives. This benefit can be reciprocal as well—pain research might also inspire new machine learning algorithms for single-shot and transfer learning, for example^32^.

The machine learning field is quickly changing and becoming a central tool for many scientific disciplines—in fact, multiple studies cited in other sections of this review have used it in some form. In some ways, as a tool and as an academic discipline, its use and study can foster the collaborative research we advocate for throughout this paper. But pain researchers must not take for granted that machine learning is a sufficient or superior way to explain pain mechanisms or predict its occurrence, particularly in the absence of contextual knowledge of the complex nature of pain.

## Discussion

The methods overviewed here cover a wide range of possible technologies and processes that exist today in the pain field. As they move from research settings into applied settings, they have the potential to help better personalize pain treatment, increase access to pain care, improve clinical trial accuracy, and influence or transform many aspects of the digital health space. However, in additional to the promising advantages and existing strengths of these methods, each also has a considerable number of concerns, limitations, or critical issues which need to be acknowledged and addressed.

All technologies and methodologies are simultaneously situated within and productive of multi-layered contexts of creation and use. The *context of creation* represents the ideas, people, processes, and data that are brought into or created within the design, implementation, and initial analyses of a given project. In the pain field, this is largely within the domain of academic, medical, or industry research, and it includes the physical hardware (e.g., neuroimaging scanners, sensors, paper forms, smart devices), the software and digital infrastructure (e.g., algorithms, internet, data capture systems, apps), raw or preprocessed data forms (e.g., acoustic files, images, transcriptions, reported outcomes, derived features), data sources (e.g., pain patients themselves, care givers, crowd- or-open sourced datasets), and data practitioners (e.g., the researchers designing the study or collecting and analyzing the data). In contrast, the *context of use* is largely situated within medical practice and the healthcare and wellness industry complex (e.g., hospitals, pharmaceutical and medical device companies, biomedical startups, insurance companies), and it includes clinical practitioners (e.g., pain physicians or other care providers), end-users and consumers (e.g., pain patients, pain communities, care givers, clinicians, device representatives), and the application or purpose itself (e.g., monitoring, treating, communicating). Importantly, both creation and use intersect with ongoing and historical social contexts as well.

When reviewing the contexts of creation and use within pain research, four distinct but inter-related problems consistently emerge: (1) a lack of representative datasets, (2) a tendency toward scientific reductionism and essentialism, (3) harmful assumptions around scientific objectivity and associated expertise or legitimacy, and (4) the potential for unchecked application or use of findings. As we have seen, large language models and sensor datasets are known to be non-representative; the same is also true for many neuroimaging datasets (364–367). This has implications for downstream contexts of use, as it could introduce bias into models or findings, potentially making future treatment decisions unfair, exacerbating existing pain care disparities, or even resulting in harm or death. Another observation is that all methods are inherently limited in the extent to which they can adequately capture pain experience—they all run the risk of oversimplifying the pain experience, whether elevating one dimension over another or ignoring an aspect altogether. For example, in both sensor- and neuroimaging-based methods, we see researchers referring to measures as “biomarkers” of pain, which tends to reduce pain into a unimodal phenomenon, one based in or best known through underlying biology instead of psychosocial factors. Likewise numeric, language-based, and visual PROs might better capture more of the cognitive elements of pain or even some of the social dynamics but may fail to acknowledge important physiological mechanisms.

We also see a tendency for pain researchers to view pain as “an object” and patients as sources of data as opposed to experts. This reproduces unequitable power dynamics and problematic assumptions about what can be measured, what should be measured, and whose measurements matter. For example, brain features, physiological data, or facial recognition data are commonly referred to as “objective measures of pain” (368–372)^33^. This terminology positions the collected measures of the body as somehow better because they are presumed to be less subjective or more “neutral” since patient's self-report is removed. It is worth spending more time on this presumption of objectivity due to its pervasiveness across pain methods and scientific practice. As scientists, we are taught that production of knowledge is only valid if our findings are “intelligible” or known, and that almost any unknown can—through rigorous methods, quantitative metrics, a bit of intellect, and hard work—eventually be made intelligible (373). We are also taught that through the scientific method we are somehow magically able to remove ourselves from the context of our science, echoing the argument of neutrality in that “good science” is not influenced by subjective phenomena like values, opinions, experiences, or emotions. But these ideas are simply that—just ideas. They represent notions and assumptions that aren't guaranteed or necessary truths (292, 372, 374, 375). This line of reasoning and particular use of language matters for pain research not only because the notion of objectivity is completely *antithetical* to the IASP definition of pain, but also because it can create a false hierarchy of data value and expertise. The notion of objective pain metrics may work against patient self-reports by appearing less noisy, less biased, or even more trustworthy. In turn, this can result in bias against patients, missed opportunities for treatment, or potentially dangerous overtreatment if a purported “objective measure” contradicts a patient's “subjective statement.” Perhaps more fundamentally, the intersectionality of pain as a biological, psychological, social, and cultural reality that can be simultaneously hidden and seen or embodied and disembodied, calls into question whether it can even be “measured” in the first place (360, 374, 376).

This is not, of course, to say that we cannot or should not study pain but rather that as a field, we need to be critical of the assumptions of our work, the questions we ask, and the goals of our research applications. We also need to re-center pain patients in our processes and see their lived experiences as valid forms of expertise. Given all this, researchers should keep in mind that amassing multimodal data alone or through collaboration (as we suggest below) is not a solution by itself. In the end, a primary goal in pain research has been to try to impose order on an experience which has remained difficult to define, in the hopes of creating more scalable pain diagnoses and treatments. While diversifying data sources may help represent pain more realistically or wholistically, attempts to scale subjectivity and ambiguity or ignore them (i.e., pain prediction models) runs a risk of disadvantaging data outliers (376) and delegitimizing the pain of those who do not fit the model. Researchers, in seeking to use or build large data sets, should also be wary of “scale thinking,” as data amount or data diversity is not necessarily proportional to model performance (377), and can be unintentionally extractive (337).

Finally, pain research is often done without much thought to widespread application. However, many of the methods utilized, datasets and models created, or associated findings might be relevant to actors *outside* direct patient care, and this raises serious ethical questions about the use of pain data, methods, and tools. For instance, pain researchers might be comfortable if the data they collected or the predictive models they built were used to help physicians better triage pain care or reduce the likelihood of patients being exposed to detrimental medicinal side effects. However, would they still feel comfortable if the same dataset was shared with insurance companies to infer pre-existing chronic pain conditions and potentially prevent some pain patients from receiving care? Would they let their algorithms be used in legal cases to determine legitimacy of workman's compensation claims? Would they want their amassed EMA, sensor, and language data being sold to third party companies whose intentions are unknown? Would they advocate for application in the absence of robust and repeated validation? Although these concerns are limited within the academic space due to Institutional Review Board data sharing requirements, in industry research spaces where client needs and business potential might sometimes take precedence over basic research considerations, concerns around dual- or multi-use technology or repurposing of data and algorithms are particularly relevant.

So where does this leave us, as researchers, as clinicians, and as an entire field? On the one hand, there are multiple take-aways and concrete actions that individual practitioners can do to improve their research today. For example, researchers can engage in reflective and reflexive praxis to combat the positivity bias that is rampant in the pain field, due to the idea that any research that is intended to minimize pain or suffering must automatically be beneficial. Additionally, labs can engage in data practices which combat individual and systemic biases in healthcare and medical pain research, including but not limited to: using simulated or randomized data sets to blind researchers during analyses (378, 379) conducting fairness audits or applying de-biasing techniques, and aiming to collect data from a diverse and importantly *representative* set of patients to account for differences due to biology as well as socioeconomic indicators of health. The latter means fundamentally shifting some of the processes and ideas that go into creating eligibility requirements or advertising for clinical studies, as well as focusing on and dedicating resources toward building and fostering trust from and within patient communities who have been previously harmed or invalidated by medical research (380). Funding agencies may also consider allocating additional resources in grant awards so that recipients are able to adapt to patient needs in the contexts of accessibility, digital literacy, and digital equity. Researchers can also be more intentional and careful about the language used in their grants and publications. Practically, this might be as simple as being upfront and transparent about limitations like under-powered samples or degrees of freedom (381). Critically, this would look like reframing study designs, labels, and interpretations to confront systemic biases and stereotypes in pain research [for a concrete example of anti-racist pain methods, see (382)]. Regarding methodologies and applications of the findings, researchers and clinicians can take the time to do thorough informed consent discussions so that patients and participants understand the kinds of data being collected, how they are collected, and their intended use. Practitioners can also combat unintended dual-, multi-, or repeated-use or media contortion of results or outcomes *via* pre-registration (383), as well as by setting and communicating clear limits for use and advocating for validation, replication, and testing to combat hype or overselling of findings (381).

On the other hand, this review signifies the urgency to move *beyond* individuals, labs, or specific disciplinary or technical fixes, and advocates for multimodal and multidisciplinary methods. Because each method discussed contains an aspect another does not, it paves the way for interesting and innovative collaborations that combine different strengths and mitigate different weaknesses Moreover, this review shows the importance and, we'd argue, *necessity* for including pain patients and social scientists in pain research processes (from design to interpretation and application). There already exist many examples of multidisciplinary collaborations, participatory methods, and/or multimodal outcomes that pain practitioners can look toward for inspiration and re-use, including painimations (185, 186), photovoice (384), pain cards (75, 183, 385), cross-culture approaches (386, 387), and more (388). Of course, this is not to say that collaboration will be easy or straight forward. It can be challenging to know when and how to best integrate different collection methods, measurement requirements, or data types. As with each individual method, the specific research question and context (e.g., the pain condition at hand, clinical standards or procedural requirements, resource allocation, existing expertise, etc.) will all shape decisions around if and how to combine forces. This is an area of active exploration and investigation, and to date, there do not exist any guidelines or best practices for collaboration within, or integration of, pain research methods.

## Conclusions

In this paper, we aimed to not only summarize the benefits and limitations of a breadth of existing and emerging pain methods, but also show their relationships to larger sociotechnical considerations and provide points of potential intersection in techniques and measures. It is our hope that this review can contribute to the larger pain field by functioning both as a mirror and a north star *via* the identification of “pain points” and gaps in our thinking and practice, the outlining of potentially mitigative actions, and the encouragement of cross- and multi-disciplinary collaborations amongst knowledge creators and care providers. As a field, if we care about upholding and advocating for the definition of pain put forth by IASP, we cannot continue to operate under the guise of research or clinical practice as usual. We cannot continue to ignore systemic inequities in pain care, as these biases end up in our data and circle back to inform future pain practice *via* our models and results. This cycle must stop. We cannot continue to function under the assumption that some forms of data are more valid than others, that some forms of research methods are more valuable than others, or that we can treat various pain metrics as if they can be easily aggregated, combined, or compared without considerable forethought and attention to context. The transformation of the field will likely involve a balance of qualitative and quantitative practices and tools, as well as empirical and theoretical modes of inquiry, and will necessitate a need for multiple ways of thinking about, communicating, and approaching pain that break traditional disciplinary, skill, and knowledge boundaries. What the future pain field will look like remains unknown, but ultimately and critically, it is up to *all* of us to build it.

## Positionality Statement

SB is a White, cis-gender female, able-bodied, ace researcher for a tech company. Her primary research background is in cognitive neuroscience and bioethics, with 14 years in the pain field. Her epistemic and methodological lenses are influenced by patient-centered care practices and design, as well as feminist and critical disability theory. As a researcher trained in both biological and social sciences, she wants to encourage the habit of critical self-reflection and reflexivity within scientific research and the transparent sharing of backgrounds, experiences, and values that may influence how researchers approach, frame, conduct, and analyze their work. AB is a White and Filipino cis-gender male from the Midwest who has worked in tech as a data scientist for 4 years. He is a former neuroscientist with a background in theoretical neuroscience and conscious perception.

## Author Contributions

SB outlined and organized the manuscript. SB and AB co-drafted, co-wrote, and co-edited the manuscript. All authors contributed to the article and approved the submitted version.

## Conflict of Interest

SB and AB are paid employees of IBM Research.

## Publisher's Note

All claims expressed in this article are solely those of the authors and do not necessarily represent those of their affiliated organizations, or those of the publisher, the editors and the reviewers. Any product that may be evaluated in this article, or claim that may be made by its manufacturer, is not guaranteed or endorsed by the publisher.

## Footnotes

^1^International Association for the Study of Pain (IASP). *Definition of pain*. In: *Terminology* (2020). Available online at: https://www.iasp-pain.org/resources/terminology/.

^2^O'Donnell J, Alltucker K. *Medical Bias: From Pain Pills to COVID-19, Racial Discrimination in Health Care Festers*. USA Today (2020). Available online at: https://www.usatoday.com/story/news/health/2020/06/14/festering-racial-bias-health-care-factor-covid-19-disparities/5320187002/.

^3^Stein NL. *Memory for Everyday and Emotional Events* (1997). Available online at: http://site.ebrary.com/id/10806640.

^4^Some examples in Abstracts from the North American Neuromodulation Society's 2021 Virtual Meeting, January 15–16, 2021. Neuromodulation: technology at the neural interface. (2021) 24:e1–276.

^5^Barron D. *The Problem With Pain Scores*. Scientific American (2021). Available online at: https://www.scientificamerican.com/article/the-problem-with-pain-scores/.

^6^Boyd Z, Elliot Z, Fruehwald J, Hall-Lew L, Lawrence D. An evaluation of sociolinguistic elicitation methods. In: *The 18th International Conference of the Phonetic Sciences*. Glaskow (2015). Available online at: https://www.internationalphoneticassociation.org/icphs-proceedings/ICPhS2015/Papers/ICPHS0800.pdf.

^7^OpenAI. *Better Language Models and Their Implications* (2019). Available online at: https://openai.com/blog/better-language-models/.

^8^Eveleth R. *Beyond the Smiley-Face Pain Scale*. The Atlantic (2015). Available online at: https://www.theatlantic.com/health/archive/2015/01/beyond-the-smiley-face-pain-scale/384049/.

^9^Additional media coverage examples: https://www.brookings.edu/research/detecting-and-mitigating-bias-in-natural-language-processing/ and https://www.technologyreview.com/2021/05/20/1025135/ai-large-language-models-bigscience-project/.

^10^Example: painexhibit.org.

^11^Example: https://painimation.pitt.edu.

^12^Hughes V. Using Dance/Movement Therapy and Laban Movement Analysis to Build a Better Model of Rehabilitation for Chronic Pain; 2018. Available: https://digitalcommons.slc.edu/cgi/viewcontent.cgi?article=1041&context=dmt_etd.

^13^Some Examples: *Unrest, On a Scale of 1 to 10, Tipping the Pain Scale*, and *This Might Hurt*.

^14^Nicholls DA, Groven KS, Kinsella EA, Anjum RL. *Mobilizing Knowledge in Physiotherapy: Critical Reflections on Foundations and Practices* (2021). Available online at: http://search.ebscohost.com/login.aspx?direct=true&scope=site&db=nlebk&db=nlabk&AN=2628279.

^15^Rauck R. Predicting pain now and in the future through personalized physiologic mobility metrics. In: *Poster presented at; 2021 Jan 15 North American Neuromodulation Society, Virtual Meeting* (2021).

^16^See two examples: (1) Wetsman N. *Data From Health Apps Offers Opportunities and Obstacles to Researchers*. The Verge (2019). Available online at: https://www.theverge.com/2019/7/3/20681254/data-health-apps-clue-period-tracking-sleep-fitness-research. (2) Wetsman N. *Apple Watch's Data ‘Black Box' Poses Research Problems*. The Verge (2021). Available online at: https://www.theverge.com/2021/7/27/22594178/apple-watch-data-research-heart-rate-reliability.

^17^van Zalk N, Lalitharatne TD, Tan Y, Nanayakkara T. *Lack of Universality in Pain Recognition from Animated Facial Expressions*. Open Science Framework (2021). Available online at: https://osf.io/9ukqy.

^18^Nakai T, Nishimoto S. Preserved representations and decodability of diverse cognitive functions across the cortex, cerebellum, and subcortex. *Neuroscience*. (2021) [https://doi.org/10.1101/2021.12.09.471939].

^19^Example: https://www.kernel.com.

^20^Example: https://www.humanbrainproject.eu.

^21^Example: https://www.humanconnectomeproject.org.

^22^Schulz M-A, Bzdok D, Haufe S, Haynes J-D, Ritter K. Performance reserves in brain-imaging-based phenotype prediction. *Neuroscience*. (2022). [https://doi.org/10.1101/2022.02.23.481601].

^23^Reinsel D, Gantz J, Rydning J. *The Digitization of the World From Edge to Core*. IDC (Data Age 2025). Report No.: #US44413318 (2018). Available online at: https://www.seagate.com/files/www-content/our-story/trends/files/idc-seagate-dataage-whitepaper.pdf.

^24^Lum K, Isaac W. To predict and serve? *Significance Mag*. (2016) 13:14–9.

^25^Barocas S, Hardt M, Narayanan A. *Fairness and Machine Learning*. fairmlbook.org (2019). Available online at: http://ww.fairmlbook.org.

^26^For a recent example, see: https://www.wired.com/story/opioid-drug-addiction-algorithm-chronic-pain/.

^27^Fussell S. *An Algorithm That ‘Predicts' Criminality Based on a Face Sparks a Furor*. WIRED (2020). Available online at: https://www.wired.com/story/algorithm-predicts-criminality-based-face-sparks-furor/.

^28^Example: https://www.affectiva.com.

^29^Example: https://www.kaggle.com.

^30^Example: https://github.com/philippwerner/pain-database-list.

^31^Example: https://www.openpain.org.

^32^Mohamed S, Oft D. Pain and machine learning. In: *Workshop on Biological and Artificial Reinforcement Learning* (2020). Available online at: https://deepmind.com/research/publications/2020/Pain-and-Machine-Learning.

^33^Examples in media include: https://www.healthline.com/health-news/mental-scientists-objectively-measure-pain-for-the-first-time-041213, https://www.apa.org/monitor/2017/11/measure-pain, and https://www.sciencedaily.com/releases/2011/09/110913172623.ht.
